# A bibliometric analysis of speech and language impairments in Parkinson’s disease based on Web of Science

**DOI:** 10.3389/fpsyg.2024.1374924

**Published:** 2024-06-19

**Authors:** Xueyao Pan, Bingqian Liang, Ting Cao

**Affiliations:** ^1^School of Foreign Languages and Literatures, Chongqing Normal University, Chongqing, China; ^2^School of Foreign Studies, Anhui Xinhua University, Hefei, Anhui, China

**Keywords:** Parkinson’s disease, speech and language impairments, Citespace, bibliometric analysis, Web of Science database

## Abstract

Many individuals with Parkinson’s disease suffer from speech and language impairments that significantly impact their quality of life. Despite several studies on these disorders, there is a lack of relevant bibliometric analyses. This paper conducted a bibliometric analysis of 3,610 papers on speech and language impairments in Parkinson’s disease patients from January 1961 to November 2023, based on the Web of Science Core Collection database. Using Citespace software, the analysis focused on annual publication volume, cooperation among countries and institutions, author collaborations, journals, co-citation references, and keywords, aiming to explore the current research status, hotspots, and frontiers in this field. The number of annual publications related to speech and language impairment in Parkinson’s disease have been increasing over the years. The USA leads in the number of publications. Research hotspots include the mechanism underlying speech and language impairments, clinical symptoms, automated diagnosis and classification of patients with PD using linguistic makers, and rehabilitation interventions.

## 1 Introduction

Parkinson’s Disease (PD) is the second most prevalent progressive neurodegenerative disease that affects at least 2–3% of people aged 65 years and older worldwide (Poewe et al., 2017). Some people may even suffer from the early-onset Parkinson’s disease before 50 years (Mehanna et al., 2022). Although the causes of PD are still controversial, varying from gene mutations (Billingsley et al., 2018) to non-genetic risk factors, such as exposure to toxic environment (Chen et al., 2021), chronic insomnia, dietary factors and so on (Reichmann et al., 2022), it is characterized by the progressive death of dopaminergic neurons in the substantia nigra pars compacta and the accumulation of α-synuclein (Cheng et al., 2010; Alcalay et al., 2019). The main symptoms of PD are a range of motor impairments, including akinesia and bradykinesia, rest tremor, rigidity, gait disturbance, and so on (Moustafa et al., 2016; Balestrino and Schapira, 2020). Non-motor abnormalities are also observed in some patients with PD, including sensory deficits (e.g., olfactory deficits, visual disturbances, somatosensory disturbances, etc.), neuropsychiatric symptoms like depression and anxiety, cognitive decline, and autonomic dysfunction (Schapira et al., 2017; Ray and Agarwal, 2020; Ma et al., 2023).

In recent years, the speech and language impairments in patients with PD have received increasing attention. Approximately 90% of PD patients suffer from speech disorder known as hypokinetic dysarthria, involving deficits in respiration, articulation, resonance, phonation, prosody, and speech fluency even in the early period (Brabenec et al., 2017; Atalar et al., 2023). Patients with PD often exhibit insufficient respiratory support, reduced loudness, monopitch, imprecise articulation, hoarse voice, and so on (Miller, 2017; Atalar et al., 2023). These speech disorders are often attributed to movement disorder which affects the muscular control and coordination of the respiratory, phonatory, and articulatory systems. Apart from speech disorders, individuals with PD are also susceptible to non-motor language problems. The morpho-syntactic, lexical-semantic, and pragmatic aspects are observed to be impaired in PD. Patients may have deficits in comprehending complex syntactic structures like noncanonical constructions, processing *long-distance dependency*, and selecting appropriate morphological variations of verbs (Hyder et al., 2021; Henkel et al., 2023). Word-finding difficulties and semantic comprehension difficulties are also reported in PD, especially when it comes to verbs (Bayram and Akbostanci, 2018; Wagner et al., 2020).

These speech and language disorders can significantly distress patients and their family members. With increased attention to these issues, some studies have systematically examined the characteristics, pathological mechanisms, and treatments of speech-language disorders in PD. For example, Magee et al. (2019) made a comprehensive and detailed review of the speech and voice changes in PD, suggesting that speech signals can be employed as proxy markers to detect PD. Similarly, in a review made by Miller (2017), the symptoms of communication impairments in PD (e.g., voice and speech acoustic changes, linguistic processing difficulties and pausing), factors related to communication disabilities and the neural mechanisms underlying speech and language changes were systematically discussed. In addition, Montemurro et al. (2019) provided a complete portrait of the pragmatic deficit in PD by examining 107 studies, exploring the relations between pragmatic abilities and other aspects of cognitive functioning.

However, no comprehensive bibliometric analysis has yet explored the speech and language problems in PD. Bibliometric analysis, a quantitative statistical method, can analyze the published literature, reveal relationships between works, track changes in a research field and provide guidance for follow-up researches (Chen et al., 2022; Zhang et al., 2023). This study used CiteSpace software to perform a bibliometric and visual analysis concerning the speech and language problems in Parkinson’s disease, focusing on the publishing trend, authors, institutions, countries, published journals, co-cited reference, research keywords, which may be helpful for the subsequent researches.

## 2 Methods

### 2.1 Data collection

**Figure 1:**
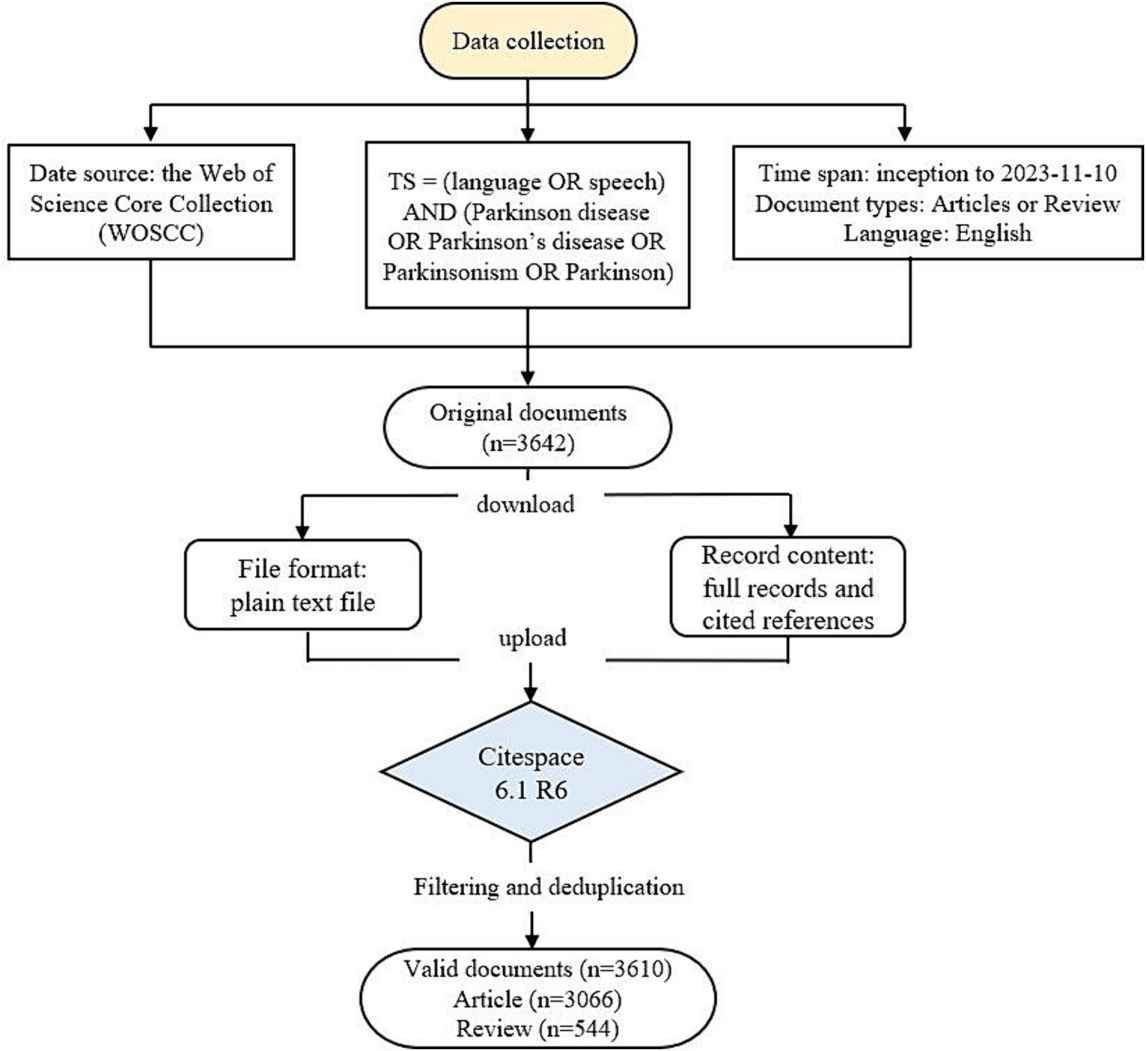
Flow chart of data collection.

Articles about PD-related speech and language problems were searched from the Web of Science Core Collection (WoSCC). Utilizing advanced search features, we systematically retrieved all publications from inception to November 10, 2023. The search strategies are as follows: TS = (language OR speech) AND (Parkinson disease OR Parkinson’s disease OR Parkinsonism OR Parkinson). Only articles and reviews were included, and other types of documents like meeting abstracts, letters, editorial material were excluded. Additionally, the publication language was limited to English. The retrieved original documents were downloaded in text format and transferred to CiteSpace 6.1 R6. for filtering and deduplication. After removing duplicates, we obtained 3,610 valid documents, including 3,066 articles and 544 reviews, for subsequent analysis (see Figure 1).

## 3 Data analysis instrument

Citespace 6.1 R6 software was used to analyze the status, trends, and hotspots in the speech and language impairments in PD. CiteSpace can integrate bibliometric analysis and systematic mapping through visual analysis methods and data mining algorithms, facilitating the identification of hotspots and research trends in specific areas. The time slice was chosen as 1 year. “Top N = 50,” and the top N % was 10%. The pruning method was “pathfinder,” “pruning sliced networks” and “pruning the merged network.”

## 4 Results

### 4.1 Analysis of annual publications

**Figure 2:**
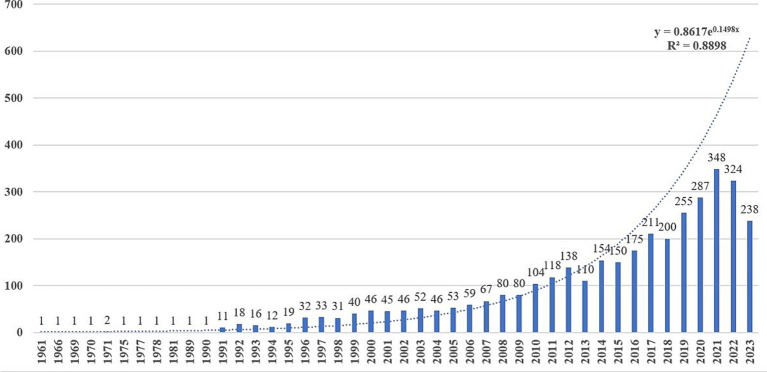
Annual publication trend of speech and language problems in PD.

The annual number of publications is depicted in Figure 2. Overall, there has been a steady increase in publications over the years, but it is not very stable. From 1961 to 2023, the number of publications increased from 1 to 238, indicating substantial growth. The first article was published in 1961, suggesting an early interest in speech-language disorders in PD. However, from 1961 to 1990, there were few publications. From 1991, the number of publications steadily rose and peaked at 348 articles in 2021, reflecting increased research interest in speech-language disorders in PD. As data retrieval ended on November 11, 2023, the number of publications for that year is incomplete. However, by fitting the curve, it is assumed that the number of publications by 2023 will continue to increase.

### 4.2 Analysis of countries/regions and institutions

**Figure 3:**
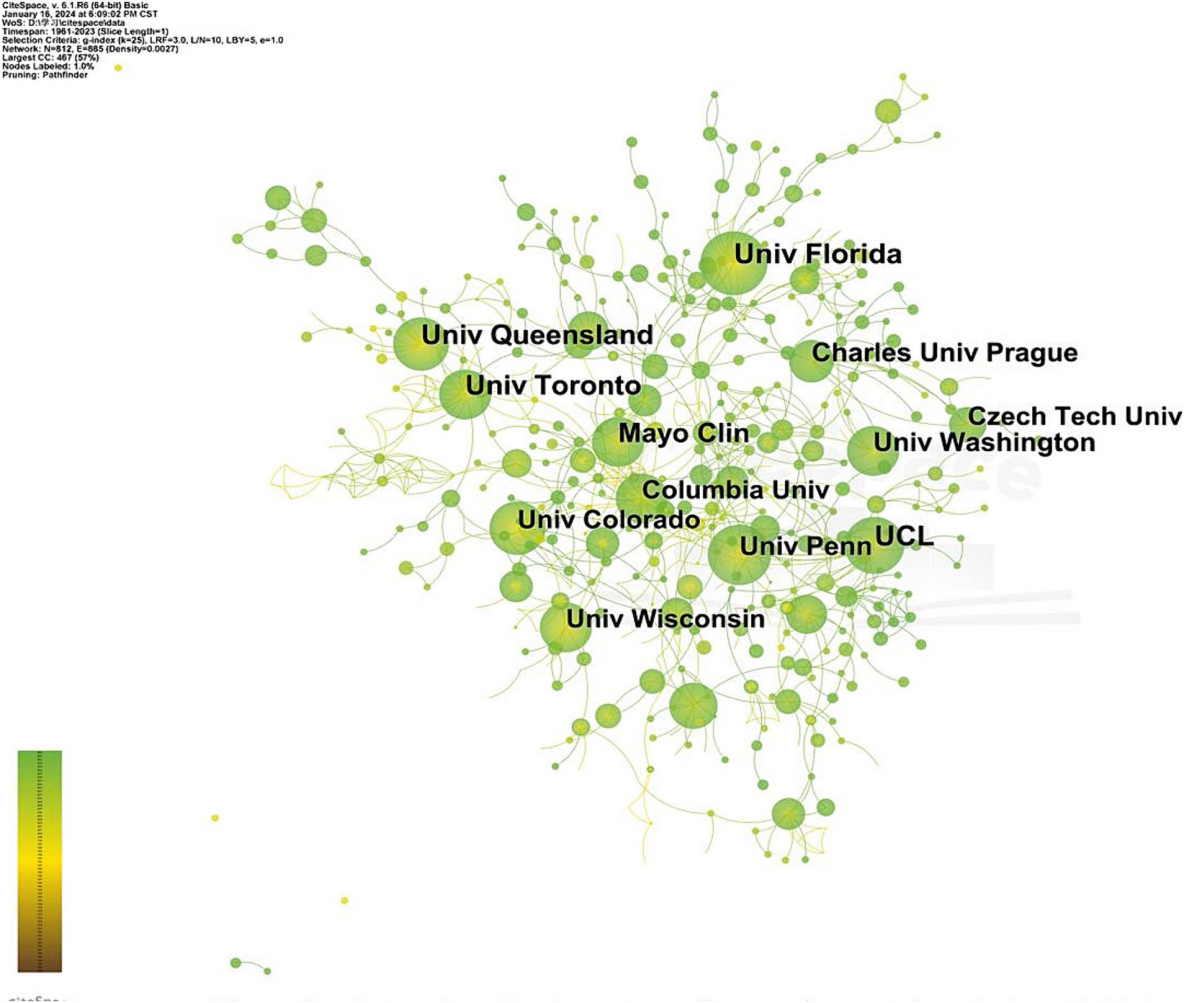
Network Visualization of countries and regions. The size of the colored nodes indicates the number of published articles. Node connections indicate the strength of the relationship between countries. The purple outer ring of the node represents the nodes betweenness centrality.

Four hundred thirteen institutions from 111 countries or regions have published articles on speech and language problems in PD. The top 10 most productive countries are listed in Table 1. As shown in Figure 3, researchers from different countries cooperate closely, forming a complex network. The USA leads in publications related the Parkinson’s speech and language impairments (1345), followed by England (367), Germany (283), Canada (262), and China (222). However, some productive countries or regions do not have high centrality. Centrality quantifies the importance of a node within a network by determining the number of shortest paths that traverse through it (Zhang et al., 2023). Singapore (0.54), Austria (0.52), and Italy (0.47) exhibit higher centrality, indicating these countries roles as bridges in research within this field.

**Table 1:** The top 10 countries/regions contributing to the *SLI in PD.*

Ranking	Country/region	Count	Centrality
1	United States	1,345	0
2	England	367	0.1
3	Germany	283	0.04
4	Canada	262	0
5	China	222	0
6	Italy	219	0.47
7	Australia	197	0
8	France	164	0.03
9	Spain	159	0.19
10	India	154	0.03

SLI, Speech-language impairments; PD, Parkinson’s disease; China, the People’s Republic of China; USA, United States of America.

**Figure 4:**
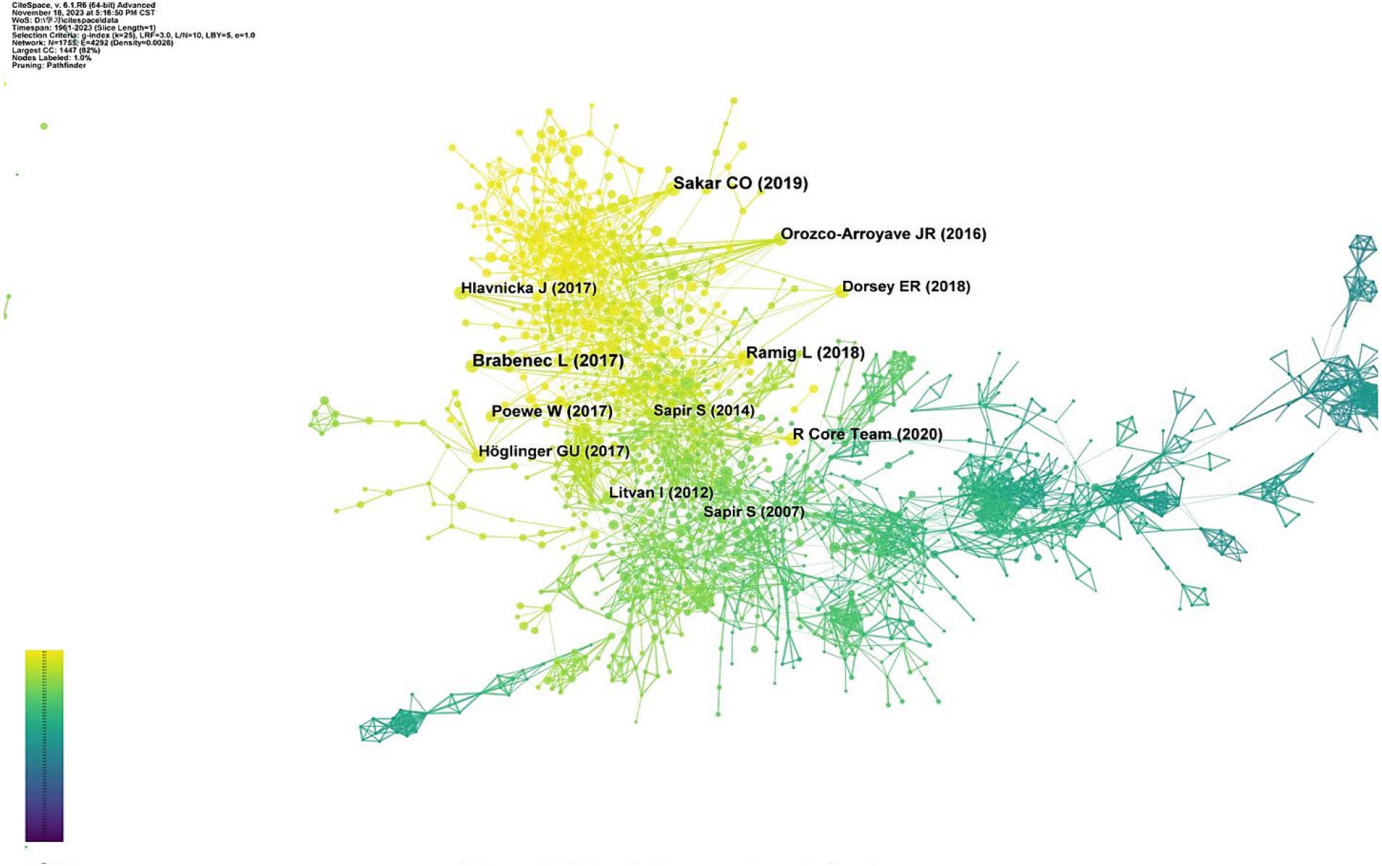
Institutional cooperation network diagram of post-stroke aphasias rehabilitation.

The top 10 most productive institutions are shown in Table 2. University College London (UCL) and University of Florida leading with 68 papers each, followed by the *Univ*ersity of *Queensland (65), Univ*ersity of *Toronto (59) and* Mayo Clin (53). The top 10 research institutions are all universities, except Mayo Clinic, indicating the leading role of universities in this field. Among the top 10 institutions, the University of Colorado, University of Pennsylvania, and UCL have relatively higher centrality, indicating their significant influence within the field. Figure 4 depicts the cooperation network among institutions, with node size representing the number of publications and line thickness reflecting the cooperation closeness between institutions.

**Table 2:** The top 10 institutions contributing to the *SLI in PD.*

Ranking	Institution	Count	Centrality
1	University College London	68	0.15
2	University of Florida	68	0.04
3	University of Queensland	65	0.04
4	University of Toronto	59	0.01
5	Mayo Clin	53	0.01
6	University of Wisconsin	49	0.05
7	Charles University in Prague	47	0.03
8	University of Colorado	46	0.31
9	Czech Technical University	44	0.04
10	University of Pennsylvania	43	0.19

SLI, Speech-language impairments; PD, Parkinson’s disease.

### 4.3 Analysis of authors

**Figure 5:**
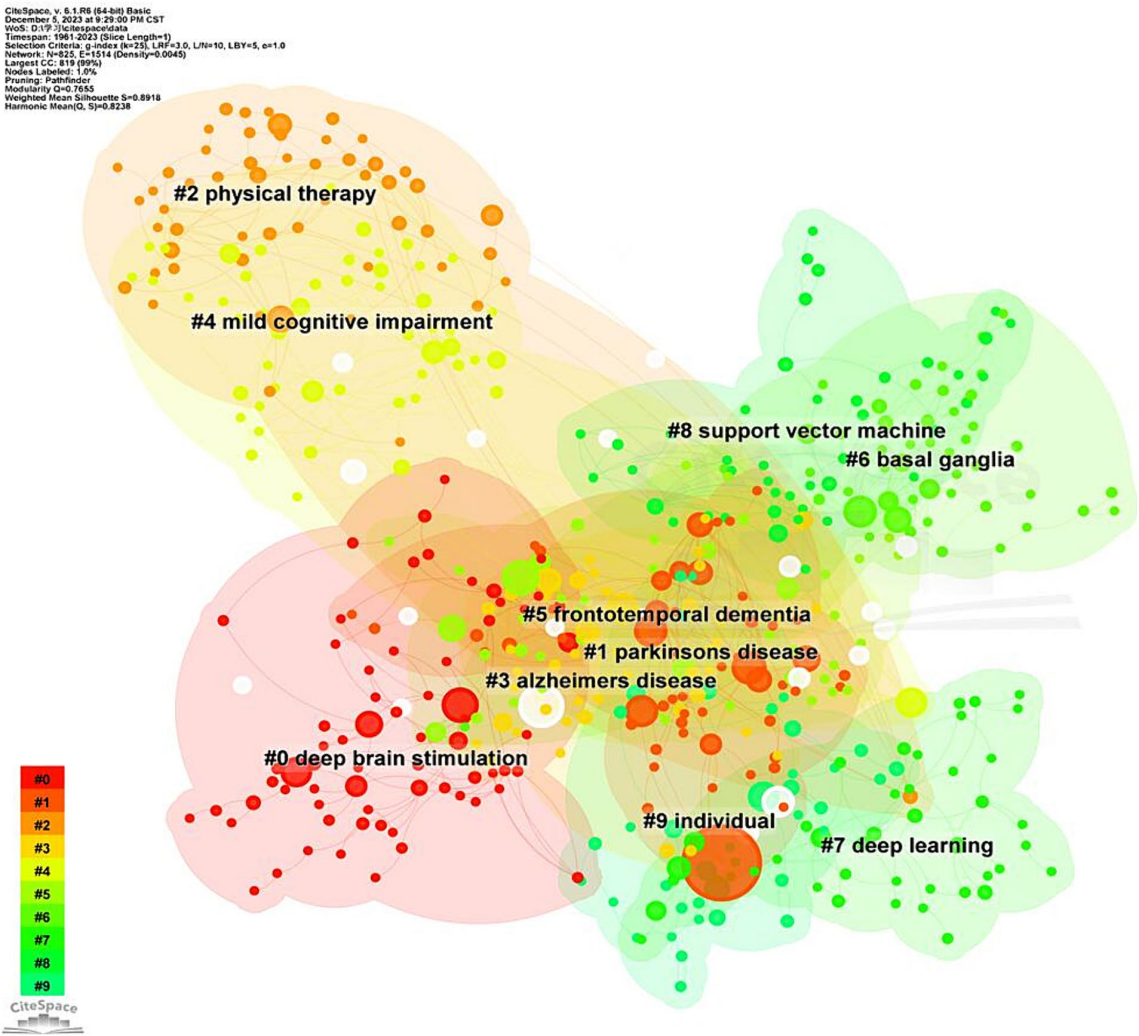
Network diagram of influential authors.

A total of 1,165 researchers have contributed to papers on speech-language problems in PD. The top 10 productive authors and co-cited authors in this field are listed in Table 3. Jan Rusz from the Czech Technical University ranked the most productive author in terms of the number of publications (42), followed by Evžen Růžička from General University Hospital (29) and Keith. A. Josephs from Mayo Clinic (26). Tereza Tykalova from Czech Technical University follows with 22 publications, and Lorraine O. Ramig from the University of Colorado-Boulder with 19. Figure 5 illustrates a collaborative network among scholars.

**Table 3:** Top 10 authors and co-cited authors of *SLI in PD.*

Rank	Authors	Count	Co-cited author	Citations
1	Rusz Jan	42	Margaret M. Hoehn	592
2	Evžen Růžička	29	Christopher G. Goetz	537
3	Keith A. Josephs	26	Aileen K. Ho	465
4	Tereza Tykalova	22	Andrew J. Hughes	408
5	Lorraine O. Ramig	19	Sabine Skodda	407
6	Skodda Sabine	19	Michael F. Folstein	406
7	Dennis W. Dickson	17	Shimon, Sapir	373
8	Michelle R. Ciucci	17	Joan A. Logemann	372
9	Kris Tjaden	16	Lorraine O. Ramig	344
10	Joseph R. Duffy	16	Florence L. Darley	344

SLI, Speech-language impairment; PD, Parkinson’s disease.

Co-cited authors are those frequently referenced together in publications, forming a co-citation bond. Margaret M. Hoehn is the most frequently co-cited author (592), followed by Christopher G. Goetz (537) and Aileen K. Ho (465). Authors with high citation and centrality levels are considered influential in their field (Su et al., 2021). Therefore, Margaret M. Hoehn, with the highest citation and centrality, may have had a significant impact in the field of speech-language problems in PD. In 1967, Hoehn and Yahr published an important study in *Neurology*, introducing the widely-used Hoehn and Yahr grading scale for evaluating PD clinical condition (Hoehn and Yahr, 1967).

### 4.4 Analysis of journals and co-cited journals

A total of 909 academic journals published papers on speech and language impairments in PD.

The top 10 journals in terms of published articles are listed in Table 4. Both *Movement Disorders* and *Neurology* contributed the largest number of publications (28), followed by *Journal of Speech Language and Hearing Research* (27), *Brain and Language* (24), *Journal of Neurology Neurosurgery and Psychiatry* (24), *Clinical Linguistics & Phonetics* (23), *Brain* (22), *Folia Phoniatrica et Logopaedica*, *Journal of Neurology*, *Journal of Voice* (each with 21 articles). Among the top 10 journals, *Brain* had the highest impact factors (IF14.5).

**Table 4:** Top 10 journals by publication frequency for SLI in PD.

Rank	Journal	Count	IF	JCR
1	Movement disorders	28	8.6	Q1
2	Neurology	28	10.1	Q1
3	Journal of Speech Language and Hearing Research	27	2.6	Q1
4	Brain and Language	24	2.5	Q2
5	Journal of Neurology Neurosurgery and Psychiatry	24	11.1	Q1
6	Clinical Linguistics and Phonetics	23	1.2	Q2
7	Brain	22	14.5	Q1
8	Folia Phoniatrica et Logopaedica	21	1	Q4
9	Journal of Neurology	21	6	Q1
10	Journal of Voice	21	2.2	Q2

IF, impact factor; SLI, Speech-language impairment; PD, Parkinson’s disease; Q, quartile category. * IF and Q in category according to Journal Citation Reports (2022).

**Figure 6:**
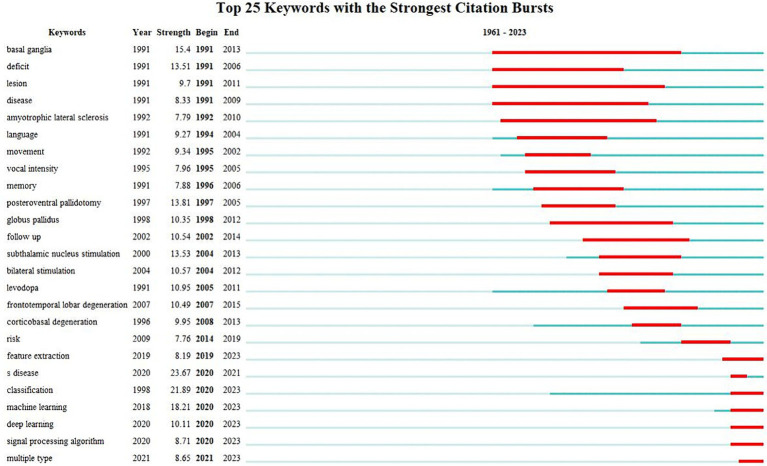
Network diagram of co-cited journals.

A co-citation analysis of journals was conducted to identify those with notable influence in a domain. *Neurology* had the highest level of co-citation (2477), followed by *Movement Disorders* (2462), *Journal of Neurology Neurosurgery and Psychiatry* (2106). *Brain* (1656) and *Parkinsonism & Related Disorders* (1346) ranked fourth and fifth, respectively, (see Table 5 for details). Among the top 10 most frequently cited journals, 80% belong to Q1. And *Lancet Neurology* has the highest impact factor (IF 48). The collaborative map of the co-cited journals of the SPL in PD is shown in Figure 6, illustrating frequent co-citations and close connections among these journals.” In the visualization, each node represents a journal that has been co-cited, and the size of the node corresponds to the frequency of co-citation for that journal. Larger nodes indicate higher co-citation frequencies. The connections between the nodes represent co-citations between the cited journals.

**Table 5:** Top 10 highly cited journal on SLI in PD.

Rank	Co-cited journal	Citations	centrality	IF	JCR
1	Neurology	2,477	0.01	10.1	Q1
2	Movement disorders	2,462	0.01	8.6	Q1
3	Journal of Neurology Neurosurgery and Psychiatry	2,106	0	11.1	Q1
4	Brain	1,656	0.05	14.5	Q1
5	Parkinsonism and Related Disorders	1,346	0.01	4.1	Q2
6	Archives of Neurology	1,089	0	7.419	Q1
7	Annals of Neurology	1,059	0	11.2	Q1
8	Lancet Neurology	993	0	48	Q1
9	Journal of Neurology	952	0.01	6	Q1
10	Brain and Language	916	0.02	2.5	Q2

IF, impact factor; SLI, Speech-language impairment; PD, Parkinson’s disease; Q, quartile category. * IF and Q in category according to Journal Citation Reports (2022).

### 4.5 Analysis of references

The top 10 most frequently co-cited references are listed in Table 6. The article by Sakar et al. (2019) published in *Applied Soft Computing* had the highest number of citations (72), followed by Brabenec et al. (2017) in *Journal of Neural Transmission* (67). Ramig et al. (2018) published in *Movement Disorder* (59) ranked the third, followed by Poewe et al. (2017) in *Nature Reviews Disease Primers (48), and* Orozco-Arroyave et al. (2016) in *The Journal of the Acoustical Society of America* (46).

**Table 6:** Top 10 references of SLI in PD.

Rank	Title	Representative Author	Year	Journal	Citation	Centrality
1	A comparative analysis of speech signal processing algorithms for Parkinson’s disease classification and the use of the tunable Q-factor wavelet transform	Okan C. Sakar	2019	Applied Soft Computing	72	0.01
2	Speech disorders in Parkinson’s disease: early diagnostics and effects of medication and brain stimulation	Lubos Brabenec	2017	Journal of Neural Transmission	67	0.02
3	Speech treatment in Parkinson’s Disease: randomized controlled trial (RCT)	Lorraine O. Ramig	2018	Movement Disorder	59	0.02
4	Parkinson disease	Werner Poewe	2017	Nature Reviews Disease Primers	48	0
5	Automatic detection of Parkinson’s disease in running speech spoken in three different languages.	J. Orozco-Arroyave	2016	The Journal of the Acoustical Society of America	46	0.06
6	Clinical diagnosis of progressive supranuclear palsy: The movement disorder society criteria	Günter U. Höglinger	2017	Movement Disorder	44	0.04
7	Automated analysis of connected speech reveals early biomarkers of Parkinson’s disease in patients with rapid eye movement sleep behavior disorder	Jan Hlavnička	2017	Scientific Reports	43	0.02
8	Global, regional, and national burden of Parkinson’s disease, 1990–2016: a systematic analysis for the Global Burden of Disease Study	Ray E. Dorsey	2018	The Lancet Neurology	41	0.02
9	Diagnostic criteria for mild cognitive impairment in Parkinson’s disease	Litvan Irene	2012	Movement Disorder	41	0.05
10	Effects of intensive voice treatment (the Lee Silverman Voice Treatment [LSVT]) on vowel articulation in dysarthric individuals with idiopathic Parkinson disease: acoustic and perceptual findings	Shimon Sapir	2007	Journal of Speech Language and Hearing	40	0.03

SLI, Speech-language impairment; PD, Parkinson’s disease.

About half of the top 10 co-cited references focused on diagnosing Parkinson’s speech disorders, paying special attention to employing advanced artificial intelligence algorithms for diagnosis. Some articles also addressed treatment methods, such as Ramig et al. (2018) and Sapir et al. (2003), which explored the effectiveness of Lee Silverman Voice Treatment for PD patients.

**Figure 7:**
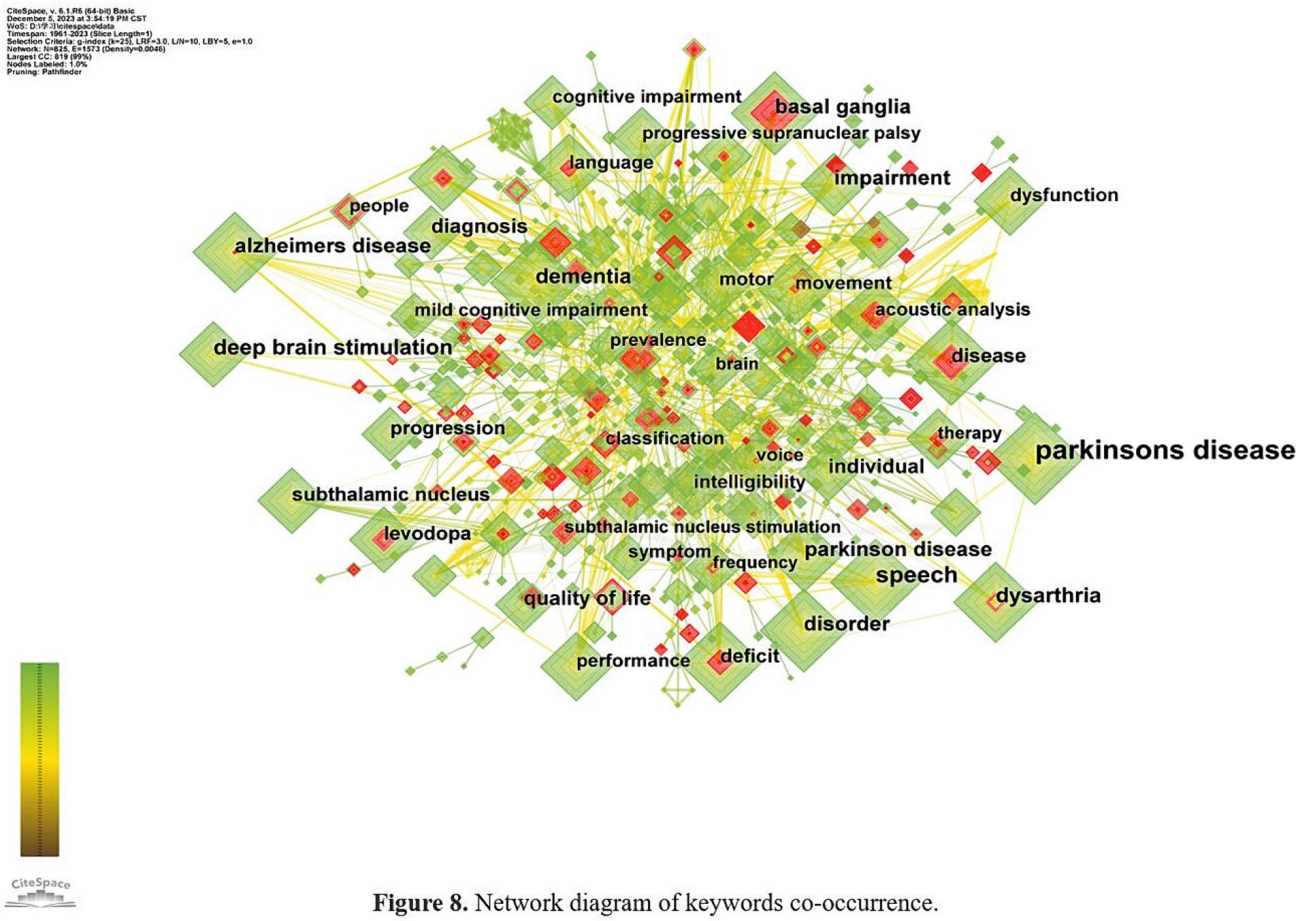
Network diagram of co-cited references.

Figure 7 highlights key studies in the field of speech and language impairments in PD and their co-cited relationships. A total of 1,729 nodes and 4,270 connections were identified in the map. Each node represents a cited reference and connections indicate co-citations.

### 4.6 Analysis of keywords

#### 4.6.1 Analysis of keywords and clusters

**Figure 8:**
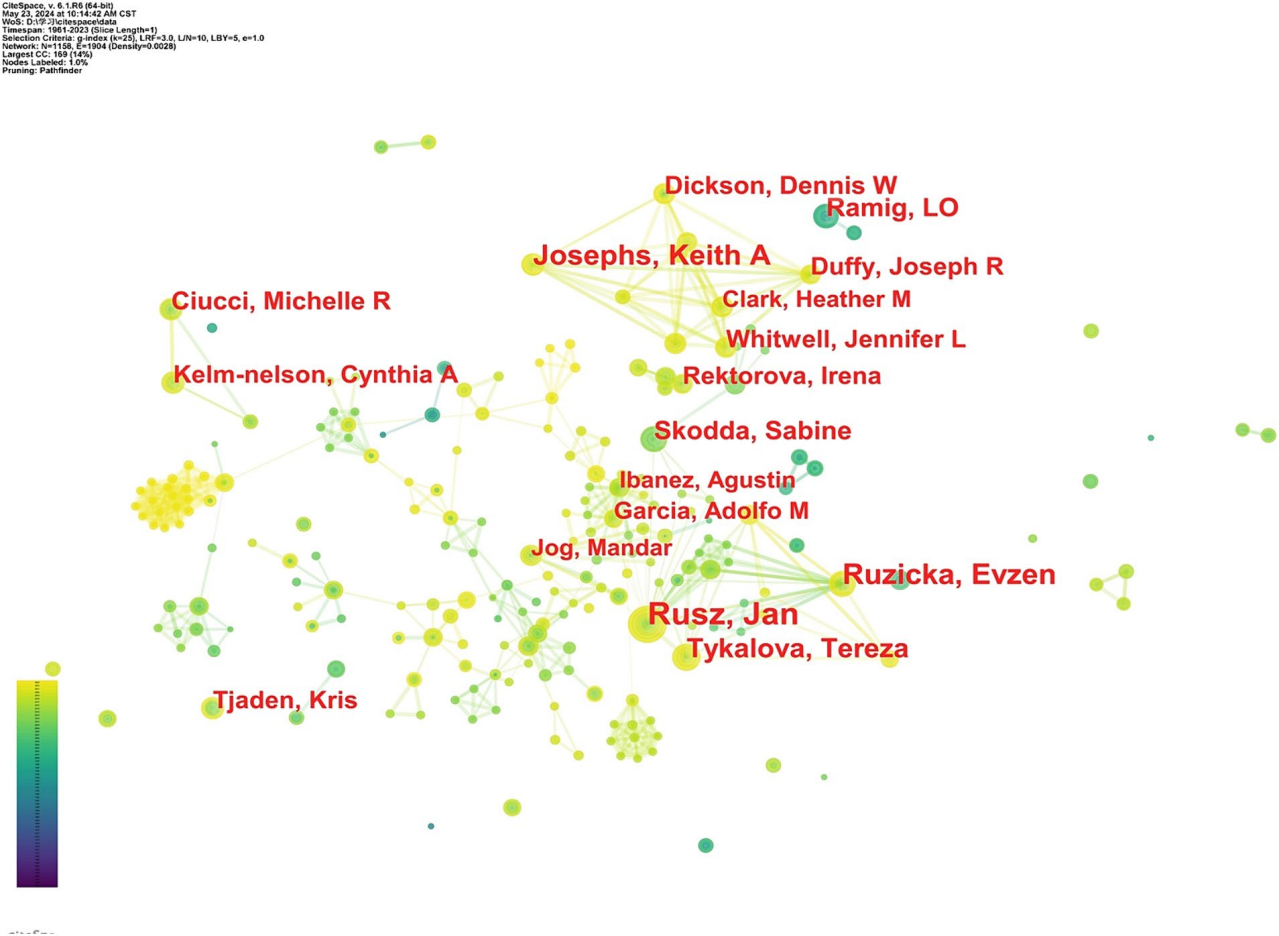
Network diagram of keywords co-occurrence.

Keyword analysis helps identify research hotpots and predict emerging trends within a given domain. The top 10 most frequent keywords are listed in Table 7, including Parkinson’s disease (1765), speech (546), deep brain stimulation (344), dementia (322), dysarthria (286), impairment (272), basal ganglia (268), disorder (248), diagnosis (241). The keyword co-occurrence network map Figure 8 shows 825 nodes and 8,145 links.

**Table 7:** Top 10 keywords for speech and language impairments in Parkinson disease.

Rank	Keywords	Count	Centrality
1	Parkinson’s disease	1765	0.03
2	Speech	546	0.05
3	Deep brain stimulation	344	0
4	Dementia	322	0.09
5	Dysarthria	286	0.08
6	Alzheimer’s disease	274	0.14
7	Impairment	272	0.1
8	Basal ganglia	268	0.11
9	Disorder	248	0.06
10	Diagnosis	241	0.07

According to the keywords, researches on speech and language impairments in PD focus on the manifestations, diagnosis and treatments of speech disorders, and the relationship between PD and other related neurodegenerative diseases.

**Figure 9:**
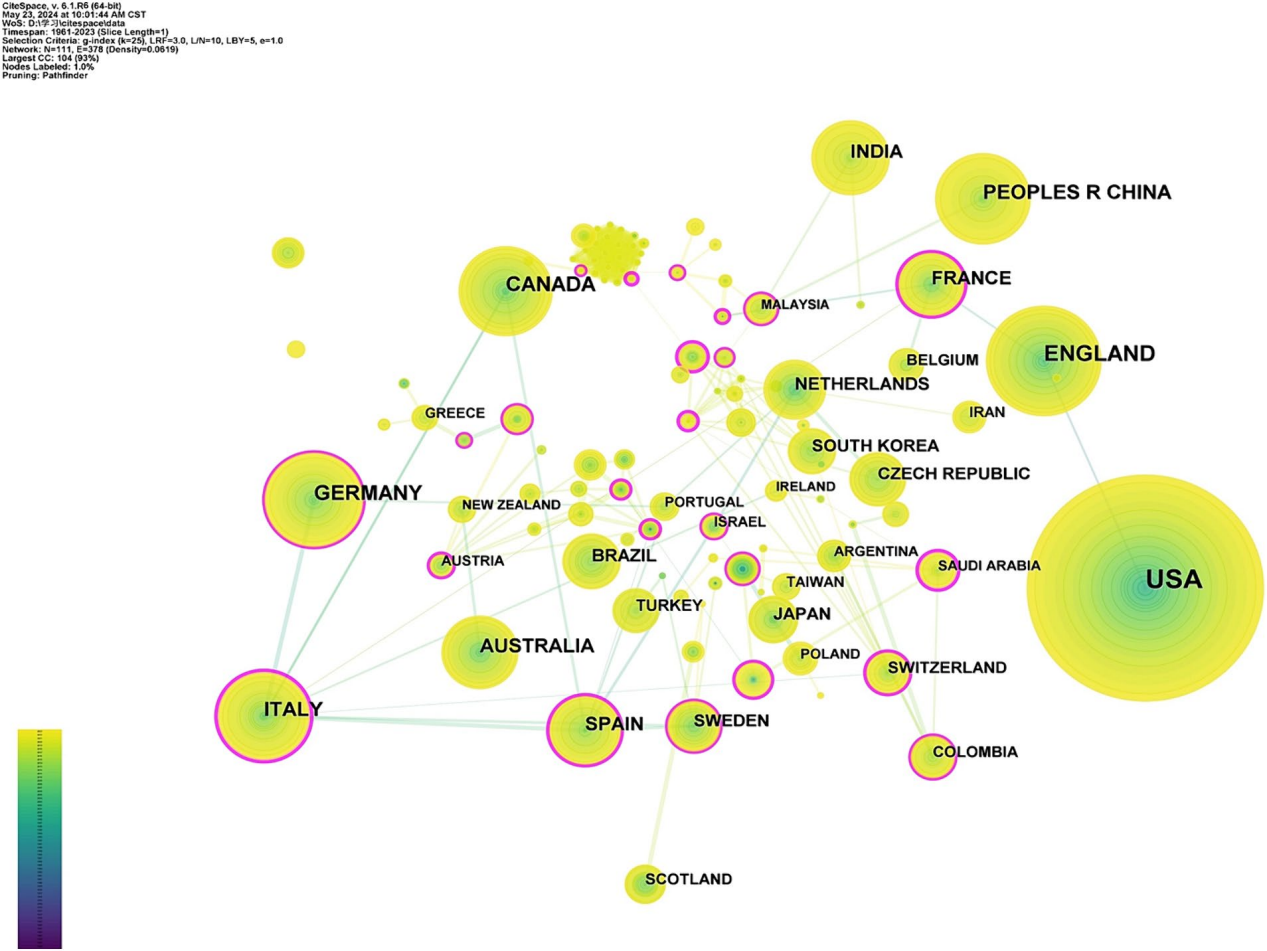
High-frequency Keywords cluster map of related literature. The nodes in the diagram represent references. Nodes with the same color are located in the same cluster, which means they belong to the same cluster topic.

The keyword clusters depict the structural characteristics among clusters and important connections within them (Qin et al., 2020). Using LLR for co-occurrence analysis, we identified 27 clusters, and the first 10 clusters were analyzed. Figure 9 presents the keyword clusters map, with the *Q* value (cluster module value) of 0.7519 (>0.3) and the *S* value (average profile value) of 0.8909, indicating a significant cluster structure and high consistency among cluster members.

The top 10 (#0–10) keyword clusters are listed in Table 8. These clusters reveal that the research topics can be divided into the following categories. The first category, including clusters #0, #1 and #5, primarily explores the diagnosis and intervention of the speech and language impairments in PD. The second category, including clusters #3, #6, #7 and #9, mainly focuses on the symptoms of PD’s speech and language impairments. The third category, including clusters #2, #4 and #8 primarily investigates the relationship between Parkinson’s disease and other cognitive disorders.

**Table 8:** Top 10 keywords clusters of SLI of PD.

Cluster ID	Size	Silhouette	Cluster name	Cluster labels (LLR)
#0	68	0.923	Deep brain stimulation	Subthalamic nucleus; quality of life; subthalamic nucleus stimulation; non-motor symptoms
#1	66	0.936	Machine learning	Occupational therapy; feature extraction; physical therapy; systematic review
#2	56	0.815	Mild cognitive impairment	Diagnosis; Parkinson’s disease dementia; dementia; risk
#3	54	0.754	Frequency	Large sample; auditory feedback; sensorimotor adaptation; response
#4	53	0.919	Cognitive impairment	Alzheimer’s disease; impairment; dementia
#5	53	0.912	Basal ganglia	Deep brain stimulation; subthalamic nucleus; language; positron emission tomography
#6	46	0.907	Syntax comprehension	Language production; performance; executive function; embodied cognition
#7	45	0.924	Parkinson’s disease	Acoustic analysis; dysarthria; voice
#8	40	0.878	Frontotemporal dementia	Corticobasal degeneration; frontotemporal lobar degeneration; tdp-43
#9	38	0.813	Movement	Speaker; multiple sclerosis; verbal fluency; pink1

LLR, log-likelihood test; SLI, Speech-language impairment; PD, Parkinson’s disease.

#### 4.6.2 Analysis of keywords with the strongest citation

**Figure 10:**
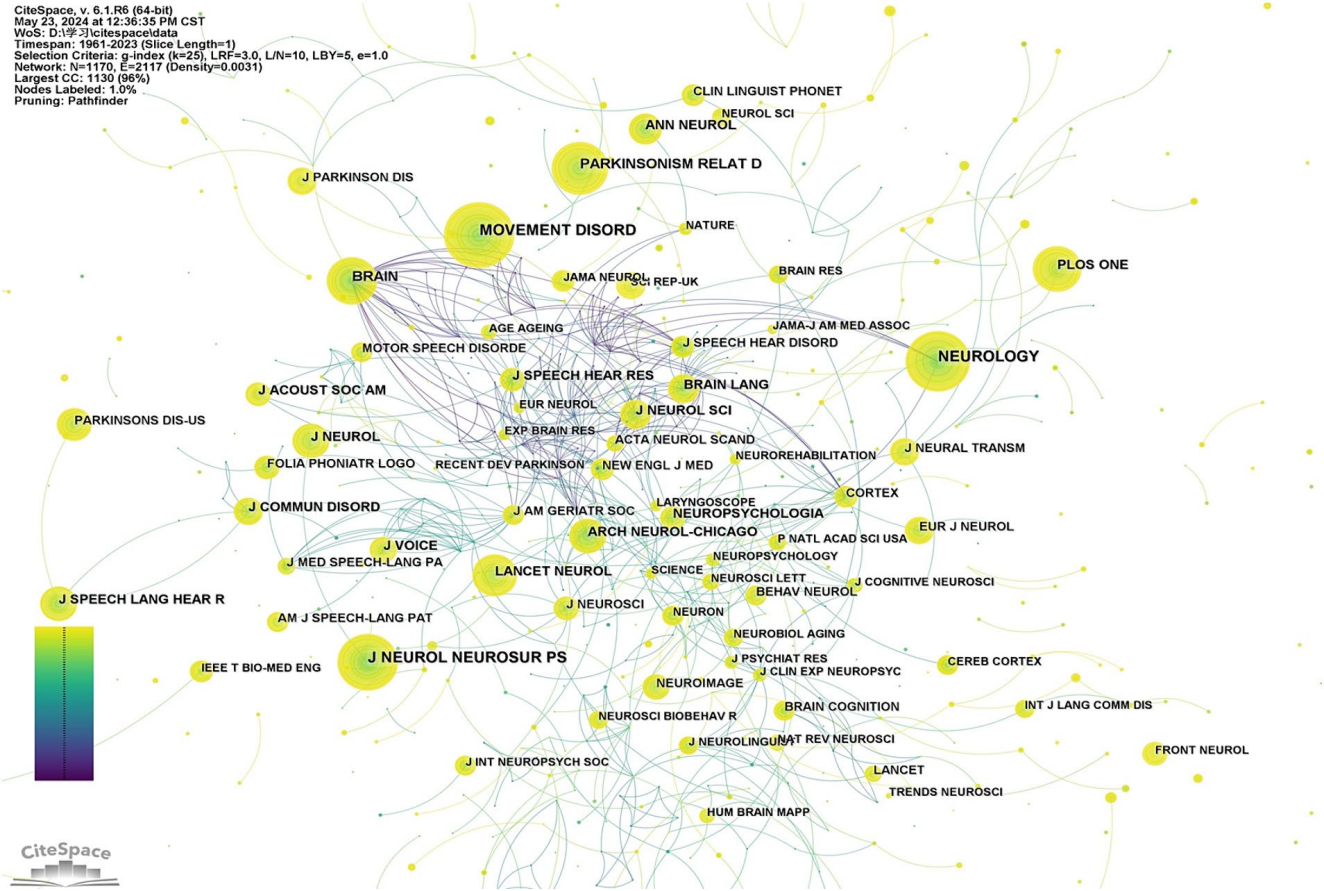
Keywords citation bursts analysis of related literature. The red line shows the time frame during which the keyword bursts were discovered, while the blue line shows the time interval.

The study of keywords citation bursts can provide insight into the research hotspots within a particular field during a specific period. Figure 10 presents the top 25 burst keywords related to speech and language impairments in PD. “Begin” and “End” signify the onset and conclusion of the burst. It can be seen that keywords with citation bursts first appeared in 1991. “Strength” refers to the intensity of the burst and indicates the believability over time (Luo et al., 2021). The 5 keywords with the highest burst strength are “Parkinson’s disease,” “classification,” machine learning,” “basal ganglia” and “posteroventral pallidotomy.”

The analysis of keyword citation bursts can be divided into three phases. The first phase (1991–2002) focus on “basal ganglia,” “deficit,” “lesion,” “disease,” “amyotrophic lateral sclerosis,” “language,” “movement,” “vocal intensity,” “memory,” “posteroventral pallidotomy,” and “globus pallidus.” This phase mainly investigates the underlying mechanism and clinical manifestations of speech and language impairments in PD. For example, Parkinson’s disease is characterized by a decline in dopaminergic innervation within the basal ganglia, resulting in motor and non-motor symptoms (Neumann et al., 2018). In addition, motor disruptions in PD are believed to originate from heightened activity in the GABAergic internal segment of the globus pallidus (Lozano and Lang, 1998), leading to hypokinetic dysarthria characterized by decreased intensity, articulatory imprecision and so on (Walsh and Smith, 2012). The second phase (2002–2014) saw burst keywords focusing on interventions for speech and language impairments in PD, including “subthalamic nucleus stimulation,” “bilateral stimulation,” “levodopa” and conditions like “frontotemporal lobar degeneration” and “corticobasal degeneration.” “Subthalamic nucleus stimulation” (13.53) became the keyword with the highest burst intensity at this stage, indicating that this intervention plays an important role in improving speech and language ability in PD patients. The third phase (2014–2023) focuses on utilizing artificial intelligence for identifying PD patients, with the primary burst keywords such as “feature extractions,” “classification,” “machine learning,” “deep learning,” “signal processing algorithm” being included. Many studies aimed to employ deep learning models to detect PD using patients’ speech signals. For example, accurate classification of PD patients can be achieved by analyzing voice features such as fundamental frequency and amplitude perturbation using techniques like time-frequency representation (TFR) and Convolutional Neural Network (CNN) (Eyigoz et al., 2020; Quan et al., 2022).

## 5 Discussion

This study used the WOS core collection database to search for relevant literature concerning speech and language impairments in PD. We conducted a bibliometric analysis of research in this area from 1961 to 2023 using Citespace software. This analysis sheds light on the research status, hotspots, and trends in the field.

### 5.1 Current status of research

Since 1961, the annual publication of articles on speech and language impairments in Parkinson’s disease has increased over the years, marking three distinct phases of development.

The first phase, from 1961 to 1990, with only 1 to 2 studies annually, reflecting initial investigations of patient’s speech and language impairments. Since the motor deficits including tremor, rigidity, and postural instability are more pronounced in PD, speech and language issues were often viewed as secondary to motor or cognitive symptoms (Miller et al., 2006). The second, spanning from 1991 to 2009, witnessed a steady rise in publications, indicating a growing interest in this field with an average of 41.37 articles per year. The third phase, from 2010 to 2023, saw a significant acceleration in research activity, with annual average number of 200.86, suggesting rapid development in this field.

Analyzing the collaborative network among countries/regions and institutions reveals that some developed Western countries like the USA, Australia, Germany, England, Canada, Italy took early interest in studying speech-language disorders in PD, resulting in a substantial volume of publications. In contrast, research in this field started relatively late in some Asian countries, especially in some developing nations, such as China (2002), Malaysia (2011), Pakistan (2014), Philippines (2017), possibly indicating differing priorities regarding patient quality of life.

Among the most prolific authors, Jan Rusz and Evžen Růžička collaborated extensively, investigating the characteristics of speech impairments in PD and developing automatic detection methods based on patients’ speech signals (e.g., Orozco-Arroyave et al., 2016; Rusz et al., 2022). Notably, *Movement Disorders* and *Neurology* emerged as the top published and cited journals, respectively, underscoring their focus on clinical neurology and their international influence. This suggests a relatively high research quality concerning speech and language impairments in PD.

### 5.2 Research hotspots and trends of SLI in PD

Keyword co-occurrence and clusters serve as indicators of popular topics within this field, and keyword bursts may highlight the research trends (Chen and Song, 2019). In addition, by analyzing publications with high citation frequency and centrality, we can also see the primary focus and frontiers in this filed. By examining keyword co-occurrence, clusters, bursts, and co-citation references, we can conclude that the research focus and frontier in the study of speech and language impairments in PD encompass several aspects.

Firstly, the mechanism underlying speech and language impairments in Parkinson’s disease has always been a research hotspot Keywords such as “dementia,” “Alzheimer’s disease,” “basal ganglia,” “subthalamic nucleus,” “levodopa,” “progressive supranuclear palsy,” and “alpha-synuclein” indicate the effort in mechanism research. Parkinson’s disease (PD) is a common neurodegenerative disease which shares some similarities to Alzheimer’s disease (AD) degenerative supranuclear palsy and other degenerative diseases. Traditionally PD has been linked to dopaminergic deficiencies. As dopaminergic neurons progressively degenerate and Lewy bodies form in the affected regions of the brain dysfunction in the basal ganglia control circuit occurs which leads to the onset of PD clinical symptoms such as hypokinesia bradykinesia of speech-organ movements and rigidity of the laryngeal muscles (Magee et al., 2019; Latif et al., 2021). Moreover, PD is also related to the accumulation of α-synuclein. For example compared with healthy controls an increased aggregated α-synuclein in erythrocytes membrane was observed in patients with PD (Yang et al., 2023). Abnormal aggregation of α-syn can lead to a series of pathological changes such as degeneration and death of dopaminergic neurons disruption of the blood–brain barrier and immuno-inflammatory reactions (Lane et al., 2015). Recently some researchers even propose that PD and AD represent different manifestations of a single condition termed “Neurodegenerative Elderly Syndrome (NES).” Lifestyle genetics and environmental factors may contribute to the divergence between these diseases (Caligiore et al., 2022).

Secondly, clinical symptoms of speech and language impairments in PD have been a focal point of research. PD is more than a motor disorder, speech and language deficits can also be observed. Patients usually suffer from perceptual motor speech deficits, manifesting imprecise consonants, reduced loudness, mono-pitch, harsh voice, reduction of intonation, altered speech intensity, inappropriate silences and so on (Magee et al., 2019). A large number of studies have taken a close look at speech disorders in PD patients, indicating that many acoustic features such as Jitter, Shimmer, Harmonics-to-Noise Ratio (HNR), tVSA (Time-Varying Spectral Analysis) can be used as “proxy markers” of disease state (Rusz et al., 2011). Meanwhile, it has been found PD patients also have non-motor language deficits, like word finding difficulties, syntax comprehension deficits. It is interesting to see that patients with PD have severe action-language impairments, like deficit of verb generation or comprehension, impaired motor-language coupling (García and Ibáñez, 2014). Some researchers hold that such impairments can be explained by the theory of embodied cognition. That is to say, the motor circuits are not only responsible for the motor control but also involved in action-language processing. Since the motor circuits are impaired in patients with PD, they can hardly process the action-language (Birba et al., 2017).

Thirdly, with the advancement of artificial intelligence, the automated diagnosis and classification of patients with PD has become key research trends. Keywords such as “diagnostic criteria,” “machine learning,” “feature selection,” and “deep learning” indicate a focus on more efficient diagnostic methods. Traditionally, clinical diagnosis often occurs long after significant neurophysiological damage has taken place, with motor symptoms such as bradykinesia, rest tremor, and rigidity being key indicators (Jankovic, 2008; Brooks, 2012). However, Parkinson’s disease also involves non-motor symptoms like hyposmia, sleep disorders, prominent apathy, depression, pain and somatosensory disturbances which should also be taken into account when diagnosing this disease (Tolosa et al., 2021). Researchers strive to identify sensitive early markers and find that changes in speech and language, including Harmonics-to-Noise Ratio (HNR), Fundamental Frequency (F0), Detrended Fluctuation Analysis (DFA), can be used as preclinical markers of PD (Amato et al., 2023). Using these linguistic makers, with the help of machine learning and deep learning techniques, we can achieve an efficient and accurate diagnosis of PD. For example, in the top 10 co-cited references, the paper by Sakar et al. (2019) obtaining the most citations, applied the tunable Q-factor wavelet transform (TQWT) to the voice signals of PD patients for feature extraction. And the paper by Orozco-Arroyave et al. (2016) proposed a method for characterizing speech signals through the automatic segmentation of utterances into voiced and unvoiced frames.

In addition, rehabilitation interventions such as “deep brain stimulation,” “subthalamic nucleus stimulation,” “bilateral stimulation” and “intensive voice treatment” have become research hotspots in recent years. Since dopamine reduction is one of the main factors causing Parkinson’s disease, traditional treatment is dominated by dopaminergic treatment. However, the effect of this treatment on speech and language impairments in PD remains controversial. Some evidence indicated that Levodopa (L-Dopa) is helpful in improving patients’ speech intelligibility and pitch control (Novotný et al., 2020; Rusz et al., 2021), while some research failed to validate of the effectiveness of the treatment (Pinho et al., 2018). However, neurosurgical therapies like deep brain stimulation (DBS) have shown more promise. Subthalamic nucleus (STN) and globus pallidus interna (GPi) are preferred DBS targets for PD treatment. DBS can modulate the firing patterns within the hyperdirect projections from motor cortical areas, as well as within the afferent and efferent fibers to the motor subthalamic nucleus (STN) (Macerollo et al., 2020), which then improves the loudness and inter-pause speech duration in PD (Magee et al., 2019). In addition, behavioral treatments also remain popular. The Lee Silverman Voice Treatment (LSVT), developed in 1987, is effective in improving loudness in PD patients. This intensive clinician delivered speech therapy can boost vocal intensity by elevating subglottic air pressure, refining vocal fold closure, and enhancing vibration through respiratory function (Ramig et al., 2018).

### 5.3 Limitations

This study used CiteSpace 6.1.R6 software for a bibliometric analysis of publications on speech and language impairments in PD. However, there are some limitations. Firstly, we only analyzed English literature in the Web of Science core collection database, which could may led to incomprehensive coverage of literature sources. Future research could include literature from CNKI, Scopus, and various other databases. Secondly, CiteSpace is a relatively simple visualization tool. In future research, we can combine more tools like VOSviewer to conduct a more in-depth and comprehensive analysis in this field.

## 6 Conclusion

This study used Citespace 6 software to visualize and analyze the literature on speech and language impairments in Parkinson’s disease (PD) from 1961 to 2023 in the web of science core collection database, this analysis reflects the current research status, research hotspots, and future development trends in this field in an intuitive, efficient, and scientific manner.

The study shows rapid development in research on speech and language impairments in PD, with increasing collaboration among scholars, institutions, and countries. Key research areas include the mechanism, the clinical symptoms, the rehabilitation of speech and language impairments in PD. Future directions involve identifying early linguistic markers of PD and using machine learning and deep learning techniques to achieve automatic diagnosis. This will require closer collaboration across molecular biology, genetics, psychology, computer science, and linguistics.

## Data availability statement

The original contributions presented in the study are included in the article/supplementary material, further inquiries can be directed to the corresponding author.

## Author contributions

XP: Conceptualization, Data curation, Formal analysis, Funding acquisition, Methodology, Software, Visualization, Writing – original draft. BL: Data curation, Software, Validation, Visualization, Writing – review & editing. TC: Formal analysis, Investigation, Project administration, Supervision, Writing – review & editing.

## References

[ref1] Alcalay R. N. Mallett V. Vanderperre B. Tavassoly O. Dauvilliers Y. Wu R. Y. J.. (2019). SMPD1 mutations, activity, and alpha-synuclein accumulation in Parkinson's disease. Mov. Disord. 34, 526–535. doi: 10.1002/mds.27642, PMID: 30788890 PMC6469643

[ref1003] Amato F. Saggio G. Cesarini V. Olmo G. Costantini G. (2023). Machine learning- and statistical-based voice analysis of parkinson’s disease patients: a survey. Expert Sys. Appli. 219:119651. doi: 10.1016/j.eswa.2023.119651

[ref2] Atalar M. S. Oguz O. Genc G. (2023). Hypokinetic dysarthria in Parkinson’s disease: A narrative review. Med. Bull. Sisli Etfal Hosp. 57, 163–170. doi: 10.14744/SEMB.2023.29560, PMID: 37899809 PMC10600629

[ref3] Balestrino R. Schapira A. (2020). Parkinson disease. Eur. J. Neurol. 27, 27–42. doi: 10.1111/ene.14108 31631455

[ref4] Bayram E. Akbostanci M. C. (2018). Neural foundations of action-related language: studies in Parkinson's disease. Turk. J. Neurol. 2018, 3–12. doi: 10.4274/tnd.04796

[ref5] Billingsley K. Bandres-Ciga S. Saez-Atienzar S. Singleton A. (2018). Genetic risk factors in Parkinson’s disease. Cell Tissue Res. 373, 9–20. doi: 10.1007/s00441-018-2817-y, PMID: 29536161 PMC6201690

[ref6] Birba A. García-Cordero I. Kozono G. Legaz A. Ibáñez A. Sedeño L.. (2017). Losing ground: Frontostriatal atrophy disrupts language embodiment in Parkinson’s and Huntington’s disease. Neurosci. Biobehav. Rev. 80, 673–687. doi: 10.1016/j.neubiorev.2017.07.011, PMID: 28780312

[ref7] Brabenec L. Mekyska J. Galaz Z. Rektorova I. (2017). Speech disorders in Parkinson’s disease: early diagnostics and effects of medication and brain stimulation. J. Neural Transm. 124, 303–334. doi: 10.1007/s00702-017-1676-0, PMID: 28101650

[ref8] Brooks D. J. (2012). Parkinson's disease: diagnosis. Parkinsonism Relat. Disord. 18, S31–S33. doi: 10.1016/S1353-8020(11)70012-8 22166447

[ref9] Caligiore D. Giocondo F. Silvetti M. (2022). The neurodegenerative elderly syndrome (NES) hypothesis: Alzheimer and Parkinson are two faces of the same disease. IBRO Neurosci. Rep. 13, 330–343. doi: 10.1016/j.ibneur.2022.09.007, PMID: 36247524 PMC9554826

[ref10] Chen J.-W. Du S.-H. Chen T.-C. Zhu K. (2022). Research hotspots and trends of exercise on Parkinson's disease: a global bibliometric analysis from 2012 to 2021. Front. Hum. Neurosci. 16:908049. doi: 10.3389/fnhum.2022.908049, PMID: 35693536 PMC9184738

[ref11] Chen C. Song M. (2019). Visualizing a field of research: A methodology of systematic scientometric reviews. PLoS One 14:e0223994. doi: 10.1371/journal.pone.0223994, PMID: 31671124 PMC6822756

[ref12] Chen Y. Sun X. Lin Y. Zhang Z. Gao Y. Wu I. X. (2021). Non-genetic risk factors for Parkinson’s disease: an overview of 46 systematic reviews. J. Parkinsons Dis. 11, 919–935. doi: 10.3233/JPD-202521, PMID: 33814465 PMC8461677

[ref13] Cheng H. C. Ulane C. M. Burke R. E. (2010). Clinical progression in Parkinson disease and the neurobiology of axons. Ann. Neurol. 67, 715–725. doi: 10.1002/ana.21995, PMID: 20517933 PMC2918373

[ref15] Eyigoz E. Courson M. Sedeño L. Rogg K. Orozco-Arroyave J. R. Nöth E.. (2020). From discourse to pathology: automatic identification of Parkinson's disease patients via morphological measures across three languages. Cortex 132, 191–205. doi: 10.1016/j.cortex.2020.08.020, PMID: 32992069 PMC7655620

[ref16] García A. M. Ibáñez A. (2014). Words in motion: motor-language coupling in Parkinson’s disease. Transl. Neurosci. 5, 152–159. doi: 10.2478/s13380-014-0218-6

[ref17] Henkel J. Hartmann C. Niccolai V. van de Vijver R. Schnitzler A. Biermann-Ruben K. (2023). Reduced syntactic recursion in spontaneous speech of Parkinson's disease patients. Acta Psychol. 236:103931. doi: 10.1016/j.actpsy.2023.103931, PMID: 37148642

[ref18] Hoehn M. M. Yahr M. D. (1967). Parkinsonism: onset, progression and mortality. Neurology 17, 427–442. doi: 10.1212/WNL.17.5.427 6067254

[ref19] Hyder R. Jensen M. Højlund A. Kimppa L. Bailey C. J. Schaldemose J. L.. (2021). Functional connectivity of spoken language processing in early-stage Parkinson’s disease: an MEG study. Neuro Image: Clinical 32:102718. doi: 10.1016/j.nicl.2021.102718 PMC8403765 34455187

[ref20] Jankovic J. (2008). Parkinson's disease: clinical features and diagnosis. J. Neurol. Neurosurg. Psych. 79, 368–376. doi: 10.1136/jnnp.2007.131045 18344392

[ref21] Lane R. K. Hilsabeck T. Rea S. L. (2015). The role of mitochondrial dysfunction in age-related diseases. Biochimica et Biophysica Acta (BBA)-Bioenergetics 1847, 1387–1400. doi: 10.1016/j.bbabio.2015.05.021, PMID: 26050974 PMC10481969

[ref22] Latif S. Jahangeer M. Razia D. M. Ashiq M. Ghaffar A. Akram M.. (2021). Dopamine in Parkinson's disease. Clin. Chim. Acta 522, 114–126. doi: 10.1016/j.cca.2021.08.009 34389279

[ref23] Lozano A. M. Lang A. E. (1998). Pallidotomy for Parkinson’s Disease. Adv. Neurol. 9, 325–336. doi: 10.1016/S1042-3680(18)30268-7 9495895

[ref24] Luo H. Cai Z. Huang Y. Song J. Ma Q. Yang X.. (2021). Study on pain catastrophizing from 2010 to 2020: A bibliometric analysis via CiteSpace. Front. Psychol. 12:759347. doi: 10.3389/fpsyg.2021.759347, PMID: 34975649 PMC8718514

[ref25] Ma B. Zhang J. Cui Y. Gao H. (2023). The anatomy and clinical significance of sensory disturbance in Parkinson's disease. J. Integr. Neurosci. 22:56. doi: 10.31083/j.jin2203056, PMID: 37258441

[ref26] Macerollo A. Zrinzo L. Akram H. Foltynie T. Limousin P. (2020). Subthalamic nucleus deep brain stimulation for Parkinson’s disease: current trends and future directions. Expert Rev. Med. Devices 17, 1063–1074. doi: 10.1080/17434440.2020.1747433, PMID: 32250645

[ref27] Magee M. Copland D. Vogel A. P. (2019). Motor speech and non-motor language endophenotypes of Parkinson’s disease. Expert. Rev. Neurother. 19, 1191–1200. doi: 10.1080/14737175.2019.1649142, PMID: 31343928

[ref28] Mehanna R. Smilowska K. Fleisher J. Post B. Hatano T. Pimentel Piemonte M. E.. (2022). Age cutoff for early-onset Parkinson's disease: recommendations from the International Parkinson and Movement Disorder Society task force on early onset Parkinson's disease. Move. Disord. Clin. Pract. 9, 869–878. doi: 10.1002/mdc3.13523, PMID: 36247919 PMC9547138

[ref29] Miller N. (2017). Communication changes in Parkinson's disease. Pract. Neurol. 17, 266–274. doi: 10.1136/practneurol-2017-001635 28687681

[ref30] Miller N. Noble E. Jones D. Burn D. (2006). Life with communication changes in Parkinson's disease. Age Ageing 35, 235–239. doi: 10.1093/ageing/afj053 16540492

[ref31] Montemurro S. Mondini S. Signorini M. Marchetto A. Bambini V. Arcara G. (2019). Pragmatic language disorder in Parkinson’s disease and the potential effect of cognitive reserve. Front. Psychol. 10:1220. doi: 10.3389/fpsyg.2019.01220, PMID: 31275189 PMC6593041

[ref32] Moustafa A. A. Chakravarthy S. Phillips J. R. Gupta A. Keri S. Polner B.. (2016). Motor symptoms in Parkinson's disease: A unified framework. Neurosci. Biobehav. Rev. 68, 727–740. doi: 10.1016/j.neubiorev.2016.07.010 27422450

[ref33] Novotný M. Dušek P. Daly I. Růžička E. Rusz J. (2020). Glottal source analysis of voice deficits in newly diagnosed drug-naïve patients with Parkinson’s disease: correlation between acoustic speech characteristics and non-speech motor performance. Biomed. Sig. Process. Control 57:101818. doi: 10.1016/j.bspc.2019.101818

[ref34] Orozco-Arroyave J. R. Honig F. Arias-Londono J. D. Vargas-Bonilla J. F. Daqrouq K. Skodda S.. (2016). Automatic detection of Parkinson's disease in running speech spoken in three different languages. J. Acoust. Soc. Am. 139, 481–500. doi: 10.1121/1.4939739, PMID: 26827042

[ref35] Pinho P. Monteiro L. Soares M. F. Tourinho L. Melo A. Nóbrega A. C. (2018). Impact of levodopa treatment in the voice pattern of Parkinson’s disease patients: a systematic review and meta-analysis. Paper presented at the CoDAS 30. 10.1590/2317-1782/20182017200 30304100

[ref36] Poewe W. Seppi K. Tanner C. M. Halliday G. M. Brundin P. Volkmann J.. (2017). Parkinson disease. Nat. Rev. Dis. Primers 3:17013. doi: 10.1038/nrdp.2017.13 28332488

[ref37] Qin Y. Zhang Q. Liu Y. (2020). Analysis of knowledge bases and research focuses of cerebral ischemia-reperfusion from the perspective of mapping knowledge domain. Brain Res. Bull. 156, 15–24. doi: 10.1016/j.brainresbull.2019.12.004, PMID: 31843561

[ref38] Quan C. Ren K. Luo Z. Chen Z. Ling Y. (2022). End-to-end deep learning approach for Parkinson’s disease detection from speech signals. Biocyberne. Biomed. Eng. 42, 556–574. doi: 10.1016/j.bbe.2022.04.002

[ref39] Ramig L. Halpern A. Spielman J. Fox C. Freeman K. (2018). Speech treatment in Parkinson's disease: randomized controlled trial (RCT). Mov. Disord. 33, 1777–1791. doi: 10.1002/mds.27460, PMID: 30264896 PMC6261685

[ref41] Ray S. Agarwal P. (2020). Depression and anxiety in Parkinson disease. Clin. Geriatr. Med. 36, 93–104. doi: 10.1016/j.cger.2019.09.012 31733705

[ref42] Reichmann H. Csoti I. Koschel J. Lorenzl S. Schrader C. Winkler J.. (2022). Life style and Parkinson's disease. J. Neural Transm. 129, 1235–1245. doi: 10.1007/s00702-022-02509-1, PMID: 35606622 PMC9463300

[ref43] Rusz J. Cmejla R. Ruzickova H. Ruzicka E. (2011). Quantitative acoustic measurements for characterization of speech and voice disorders in early untreated Parkinson’s disease. J. Acoust. Soc. Am. 129, 350–367. doi: 10.1121/1.3514381, PMID: 21303016

[ref44] Rusz J. Tykalova T. Novotny M. Zogala D. Ruzicka E. Dusek P. (2022). Automated speech analysis in early untreated Parkinson's disease: relation to gender and dopaminergic transporter imaging. Eur. J. Neurol. 29, 81–90. doi: 10.1111/ene.15099, PMID: 34498329

[ref45] Rusz J. Tykalova T. Novotny M. Zogala D. Sonka K. Ruzicka E.. (2021). Defining speech subtypes in de novo Parkinson disease: response to long-term levodopa therapy. Neurology 97:e2124-e 2135. doi: 10.1212/WNL.0000000000012878, PMID: 34607922

[ref1005] Sakar C. O. Serbes G. Gunduz A. Tunc H. C. Nizam H. Sakar B. E.. (2019). A comparative analysis of speech signal processing algorithms for Parkinson’s disease classification and the use of the tunable Q-factor wavelet transform. Applied Soft Comput. 74, 255–263. doi: 10.1016/j.asoc.2018.10.022, PMID: 32818719

[ref1004] Sapir S. Spielman J. Ramig L. O. Hinds S. L. Countryman S. Fox C.. (2003). Effects of intensive voice treatment (the Lee Silverman Voice Treatment [LSVT]) on ataxic dysarthria: a case study. American J. Speech-langu. Pathol. 12, 387–399. doi: 10.1044/1058-0360(2003/085), PMID: 14658991

[ref46] Schapira A. H. Chaudhuri K. R. Jenner P. (2017). Non-motor features of Parkinson disease. Nat. Rev. Neurosci. 18, 435–450. doi: 10.1038/nrn.2017.62 28592904

[ref47] Su Z. Zhang M. Wu W. (2021). Visualizing sustainable supply chain management: A systematic Scientometric review. Sustain. For. 13:4409. doi: 10.3390/su13084409

[ref1002] Tolosa E. Garrido A. Scholz S. W. (2021). Challenges in the diagnosis of Parkinson’s disease. Lancet Neurol. 20, 385–397. doi: 10.1016/S1474-4422(21)00030-2 33894193 PMC8185633

[ref49] Wagner D. Eslinger P. J. Sterling N. W. Du G. Lee E.-Y. Styner M.. (2020). Lexical-semantic search related to side of onset and putamen volume in Parkinson’s disease. Brain Lang. 209:104841. doi: 10.1016/j.bandl.2020.104841, PMID: 32818719 PMC8189666

[ref50] Walsh B. Smith A. (2012). Basic parameters of articulatory movements and acoustics in individuals with Parkinson's disease. Mov. Disord. 27, 843–850. doi: 10.1002/mds.24888, PMID: 22729986 PMC3418799

[ref51] Neumann W. J. Schroll H. de Almeida Marcelino A. L. Horn A. Ewert S. Irmen F. (2018). Functional segregation of basal ganglia pathways in Parkinson's disease. Brain 141, 2655–2669. 30084974 10.1093/brain/awy206

[ref52] YangY.ShiM.LiuX.ZhuQ.XuZ.LiuG.. (2023). Calcium influx: an essential process by which α-Synuclein regulates morphology of erythrocytes. J. Adv. Res. doi: 10.1016/j.jare.2023.09.009 [Online ahead of print]., PMID: 37714326

[ref53] Zhang T. Yang R. Pan J. Huang S. (2023). Parkinson's disease related depression and anxiety: a 22-year bibliometric analysis (2000-2022). Neuropsychiatr. Dis. Treat. 19, 1477–1489. doi: 10.2147/NDT.S403002, PMID: 37404573 PMC10317541

